# ^18^F-Fluorination Using Tri-*Tert*-Butanol Ammonium Iodide as Phase-Transfer Catalyst: An Alternative Minimalist Approach

**DOI:** 10.3390/ph14090833

**Published:** 2021-08-24

**Authors:** Sandip S. Shinde, Kim-Viktoria Bolik, Simone Maschauer, Olaf Prante

**Affiliations:** Department of Nuclear Medicine, Molecular Imaging and Radiochemistry, Friedrich-Alexander University Erlangen-Nürnberg (FAU), D-91054 Erlangen, Germany; shinde88@gmail.com (S.S.S.); kim-viktoria.bolik@fau.de (K.-V.B.); simone.maschauer@uk-erlangen.de (S.M.)

**Keywords:** fluorination, fluorine-18, phase transfer catalyst, copper, radiopharmaceuticals, nucleophilic substitution, ^18^F-labeling

## Abstract

The ^18^F syntheses of tracers for positron emission tomography (PET) typically require several steps, including extraction of [^18^F]fluoride from H_2_[^18^O]O, elution, and drying, prior to nucleophilic substitution reaction, being a laborious and time-consuming process. The elution of [^18^F]fluoride is commonly achieved by phase transfer catalysts (PTC) in aqueous solution, which makes azeotropic drying indispensable. The ideal PTC is characterized by a slightly basic nature, its capacity to elute [^18^F]fluoride with anhydrous solvents, and its efficient complex formation with [^18^F]fluoride during subsequent labeling. Herein, we developed tri-(*tert*-butanol)-methylammonium iodide (TBMA-I), a quaternary ammonium salt serving as the PTC for ^18^F-fluorination reactions. The favorable elution efficiency of [^18^F]fluoride using TBMA-I was demonstrated with aprotic and protic solvents, maintaining high ^18^F-recoveries of 96–99%. ^18^F-labeling reactions using TBMA-I as PTC were studied with aliphatic 1,3-ditosylpropane and aryl pinacol boronate esters as precursors, providing ^18^F-labeled products in moderate-to-high radiochemical yields. TBMA-I revealed adequate properties for application to ^18^F-fluorination reactions and could be used for elution of [^18^F]fluoride with MeOH, omitting an additional base and azeotropic drying prior to ^18^F-labeling. We speculate that the *tert*-alcohol functionality of TBMA-I promotes intermolecular hydrogen bonding, which enhances the elution efficiency and stability of [^18^F]fluoride during nucleophilic ^18^F-fluorination.

## 1. Introduction

The diagnosis and quantification of various physiological and pathophysiological processes in vivo by position emission tomography (PET) have become increasingly crucial in medical research [1]. The high resolution and sensitivity of PET allow the detection of changes in cellular function or receptor densities during disease development using molecular tracers, most frequently labeled with fluorine-18. Radiolabeled biological and pharmaceutical active molecules carrying ^18^F are of increasing importance for preclinical, clinical, and nuclear medical research due to the unique properties of ^18^F, such as low β^+^-energy, long half-life (109.77 min), and the easy accessibility of no-carrier-added [^18^F]fluoride [2]. The nuclear reaction of ^18^O(p,n)^18^F in a small-scale cyclotron is the commonly applied process for the production of [^18^F]fluoride, which involves the bombardment of H_2_[^18^O]O with highly accelerated protons, and is the basis of the supply of radiopharmaceutical facilities. The extraction of [^18^F]fluoride from water uses anion-exchange columns, particularly quaternary methylammonium (QMA) cartridges, which allow the removal and recovery of H_2_[^18^O]O. The trapped [^18^F]fluoride on the cartridge is commonly eluted using an aqueous MeCN solution of a base and phase transfer catalyst (PTC), such as Kryptofix^®^2.2.2 or tetraalkylammonium bicarbonates, which are part of a conserved protocol. In the reaction vial, the water is finally removed by azeotropic drying under heating and gas flow to provide the reactive and dry [^18^F]fluoride-PTC reagent for nucleophilic labeling of target precursors [3,4].

The nucleophilic substitution (S_N_2) is a widely adopted method in ^18^F chemistry for the introduction of [^18^F]fluoride to provide important radiotracers, such as [^18^F]FP-CIT [5], [^18^F]FDG [6], [^18^F]FMISO [7], [^18^F]F-tryptophan derivatives [8], and many others. This strategy has gained significant importance in the radiopharmaceutical field, as it is not limited to aliphatic systems, but can also be applied to aromatic systems, whereas [^18^F]fluoride is always introduced to the reaction by taking advantage of the eluting agent consisting of an appropriate solvent and the PTC.

Numerous PTCs have been developed to improve radiosynthetic processes [9,10]; among them, tetraalkylammonium salts have been studied most widely due to their counter anion exchanging ability with [^18^F]fluoride and fair solubility in most organic solvents including CH_3_CN and alcohols. The S_N_Ar radiofluorination with phosphonium borane modified cartridges and tetrabutylammonium cyanide (TBACN) as an eluting agent was reported, whereas tetrabutylammonium cations serve as PTCs and the cyanide counter anion displaces [^18^F]fluoride from the cartridge [11]. Radiochemists have made efforts to skip the typical aqueous elution of [^18^F]fluoride and the following azeotropic drying steps. Recently, Aerts et al. developed the *n*-tetradecyltrimethylammonium cation (TDTMA) to avoid the azeotropic drying step before radiofluorination [12]. Tetraethylammonium (TEA) hydrogen carbonate, tosylate, or perchlorate salts were developed by Inkster et al. to avoid basic reaction conditions as well as azeotropic drying. Such PTC salts were efficiently used for both aliphatic and aromatic ^18^F-fluorination reactions [10]. Apart from tetraalkylammonium salts, various arylonium salts, such as quaternary anilinium, diaryliodonium, and triarylsulfonium, were developed to minimalize the ^18^F-labeling approach [13]. The aforementioned onium salts act as PTC as well as promoters for radiofluorinations of aliphatic and aromatic compounds. However, radiolabeling using quaternary alkylammonium [^18^F]fluoride is much faster at >80 °C; the reaction conditions typically produce by-products, such as alcohols or alkenes, due to strongly basic PTC–solvent systems.

Recent reports on alcohol-containing solvent systems for ^18^F-fluorination [14,15] or *tert*-alcohol coordinated tetraalkylammonium fluoride [16,17] demonstrated the favorable properties of such reagents in terms of reactivity, nucleophilicity and solubility in organic solvents. Furthermore, *tert*-alcohol functionalized tri-*tert*-butanolamine was reported as a promoter of S_N_2 fluorination reactions; these accelerate the nucleophilic aliphatic substitution due to the coordination between hydroxyl (-OH) and amine functionalities and fluoride [18]. Importantly, nucleophilic properties of fluorine dominate over the basicity in favor of S_N_2 substitutions [19].

**Scheme 1:**
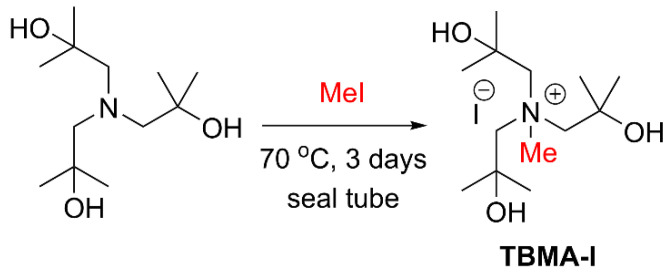
Preparation of tri-(*tert*-butanol)-methylammonium iodide (TBMA-I) from tri-(*tert*-BuOH)A.

In this work, we aimed to develop tri-(*tert*-butanol)-methylammonium fluoride as a PTC for radiofluorinations, offering reduced basicity of fluoride by hydrogen-bonding coordination. Herein, we assessed the reactivity and [^18^F]fluoride elution efficiency of tri-(*tert*-butanol)amine (tri-(*t*-BuOH)A) and quaternary ammonium salt tri-(*tert*-butanol)-methylammonium iodide (TBMA-I, Scheme 1) as PTC in the ^18^F-fluorination of 1,3-ditosylpropane and aromatic boronic acid pinacol esters as model precursors.

## 2. Results and Discussion

Initially, we examined the application of known tri-(*t*-BuOH)A [18] on the recovery of [^18^F]fluoride from QMA cartridges (Table 1). Entry 2 of Table 1 illustrates that the recovery of [^18^F]fluoride was very poor, at 11%. Most of the [^18^F]fluoride was trapped on the cartridge, similar to an elution without any PTC (entry 1). Thus, tri-(*t*-BuOH)A was converted into the corresponding quaternary ammonium form by following a reported procedure with some modifications [20,21]. Scheme 1 shows the synthesis of TBMA-I by neat-treating of tri-(*tert*-BuOH)A with 1.1 equivalents of MeI under pressure at 70 °C for 3 days. The resulting product was semi-solid and immiscible with DCM, Et_2_O, EtOAc, and hexane, but soluble in CH_3_CN, H_2_O, MeOH, and DMSO. The purity and identity of TBMA-I were confirmed by LC/MS and ^1^H-NMR analysis, and the reactivity of TBMA-I was studied by performing iodination reactions with 1,3-ditosylpropane using 1.5 equivalents of TBMA-I in CH_3_CN at room temperature for 12 h, providing the desired 3-iodo-1-tosylpropane with a yield of 67%.

With TBMA-I, we studied its property as a PTC for [^18^F]fluoride elution, as summarized in Table 1, when different parameters were optimized for the elution of [^18^F]fluoride from QMA cartridges using 1 mL of various elution mixtures of PTC. We investigated the elution efficiency of the protic functionalized quaternary ammonium iodide salt TBMA-I in an elution mixture of an aqueous solution (1 mL total volume) of K_2_CO_3_ (1 M, 15 µL) and CH_3_CN (800 µL), which showed excellent eluent properties, with an ^18^F recovery of 98.8% (entry 3). The same elution in the presence of tri-(*t*-BuOH)A resulted in a lower ^18^F recovery, similar to that in the absence of a PTC (entries 1 and 2). The concentration of TBMA-I both lower and higher than 39 µmol, did not significantly affect the efficiency of [^18^F]fluoride elution; ^18^F-recoveries of greater than 94% were obtained (Table 1, entries 4 and 5).

Entries 6–8 in Table 1 show similar elution efficiencies to the TBMA-I salt in protic solvents, such as MeOH and *tert*-BuOH, without usage of the aforementioned potassium-carbonate-containing elution solvent, producing adequate ^18^F-recoveries of 98% (MeOH) and 70% (*tert*-BuOH), respectively.

The ability of TBMA-I to facilitate nucleophilic ^18^F-fluorinations in CH_3_CN was assessed using [^18^F]fluoropropyl tosylate (**[^18^F]2a**) as a model product (Table 2). TBMA-[^18^F]fluoride was eluted from the QMA cartridge by using an elution mixture of K_2_CO_3_ (1 M, 15 µL) and H_2_O (185 µL) in CH_3_CN (800 µL), then azeotropically dried by applying anhydrous CH_3_CN (3 × 500 µL) in a gas flow at 85 °C. A solution of precursor 1,3-ditosylpropane (**1**, 6.0 mg) in anhydrous organic solvent (500 µL) was added into the reaction vial and heated at 85 °C over 20 min, and the radiochemical yield (RCY) was analyzed by radio-TLC at specific time points.

Entry 1 of Table 2 was performed using tri-(*tert*-BuOH)A, which is known to be a good promoter for fluorinations with alkali metal fluoride salts [18]. However, as mentioned above, the elution efficacy was poor using pure tri-(*tert*-BuOH)A, such that the remaining [^18^F]fluoride on the cartridge could be eluted using additional elution mixture of K_2_CO_3_ (1 M, 100 µL) in H_2_O (400 µL). Subsequently, the solvent was azeotropically dried using anhydrous CH_3_CN and a solution of precursor 1 containing additional tri-(*tert*-BuOH)A in CH_3_CN was added, but no reaction was observed. Conversely, the ^18^F-fluorination applying the conventional potassium carbonate/Kryptofix^®^2.2.2 system showed 65% RCY for the desired **[^18^F]2a**, along with a significant amount of the alcohol **[^18^F]2b** (29%) (entry 2). Similarly, the ^18^F substitution employing tetraethylammonium bicarbonate (TEAB) as PTC resulted in a 60% RCY of **[^18^F]2a** and 7% of the alcohol **[^18^F]2b** after a 20 min reaction time (entry 3). Very interestingly, the selective formation of **[^18^F]2a** was observed when the reaction was performed using TBMA-I in CH_3_CN solvent (entry 4). The same reaction applying a combination of both solvents *tert*-BuOH and CH_3_CN in a 1:4 ratio and a total volume of 500 µL offered an RCY of 50% for the desired product **[^18^F]2a**. Surprisingly, the formation of the hydrolytic byproduct **[^18^F]2b** was suppressed. Changing the solvent ratio of *tert*-BuOH and MeCN to 1:9, we found that the RCY increased to 57% of **[^18^F]2a** as a single product (Table 2, entry 6). Notably, the reaction resulted in the best conversion and chemoselectivity compared to the conventional Kryptofix^®^2.2.2-assisted reaction (Table 2, compare entry 6 with entries 2 and 3). Further evaluation of polar solvents (DMSO, DMF, and THF) indicated poor RCY (entries 7 and 8). In an attempt to find a protic solvent with an effect on the reactivity of TBMA-I, ^18^F fluorination was performed in *iso*-propanol, achieving a decreased RCY of 25% after 20 min. The similar reaction in *tert*-BuOH was attempted, but the precursor was insoluble.

These results showed that TBMA-I allows adequate recovery of [^18^F]fluoride from the QMA cartridge using the classical aqueous solution mixture and significant PTC activity in radiofluorinations.

Organic PTCs were reported for the elution of [^18^F]fluoride using MeOH as a solvent [22]. Importantly, TBMA-I has an excellent solubility in MeOH and could be used for the elution of [^18^F]fluoride without employing an additional base. Methanolic TBMA-I solutions showed a good ability to elute [^18^F]fluoride (Table 1). Furthermore, MeOH could be evaporated easily below 100 °C. These conditions are time-saving as they allow skipping azeotropic drying, which is needed after the elution under classical aqueous conditions.

Thus, we studied the reactivity of eluted [^18^F]fluoride using TBMA-I in MeOH in the absence of water and potassium salt bases. Table 3 shows the results of the radiofluorination of **1** using [^18^F]fluoride, which was eluted with a solution of methanolic TBMA-I (10.4 µmol, entry 7, Table 1), in various reaction solvent systems. Entry 1 in Table 3 was performed in CH_3_CN (4.5 mL) with *tert*-BuOH (0.5 mL) as a co-solvent when **[^18^F]2a** was afforded with 40% RCY. Varying the solvent to *t*-BuOH and to CH_3_CN ratio to 1:4 and 4:1, respectively, did not increase the RCY of **[^18^F]2a** (entries 3 and 4). We speculate that the basicity of TBMA-I is less than that of other ammonium PTCs due to the presence of three *tert*-OH moieties. Therefore, the reaction was also performed under the addition of 15 µL aqueous K_2_CO_3_ (1 M), but hardly any radiolabeled product was observed (Table 3, entry 4). To determine the solvent effect, the reaction was also carried out in pure CH_3_CN without any co-solvent, and only 14% RCY was observed for **[^18^F]2a** (Table 3, entry 5). The results suggest that the evaporation of MeOH before addition of the precursor is not sufficient, as the presence of traces of water might affect the [^18^F]fluoride reactivity. However, the elution process and the reaction conditions resulted in a maximum RCY of 40% (Table 3, entry 1), compared to the conventional *tert*-BuOH/MeCN conditions with an RCY of 57% (Table 2, entry 6), but the benefits of a simple and time-saving procedure and omitting the azeotropic drying step may offer great potential for its use in automated radiosynthesis modules.

**Figure 1:**
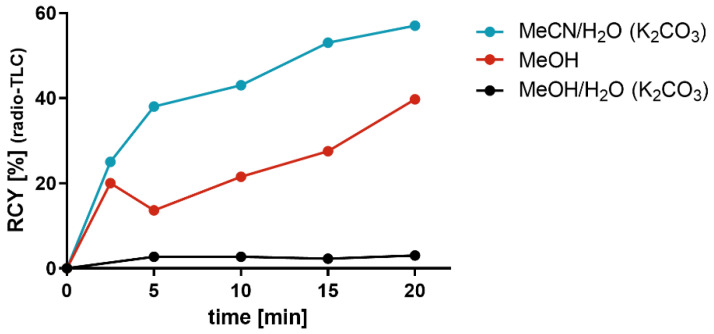
Time dependency of the RCY on the radiosynthesis of **[^18^F]2a** (Table 2) when [^18^F]fluoride was eluted from the anion-exchange cartridge by various solvent systems.

Figure 1 illustrates the comparative results of the RCY for the radiosynthesis of **[^18^F]2a** when using [^18^F]fluoride that was obtained by different elution conditions in a time-dependent manner. The reactivity of [^18^F]fluoride obtained by the classical elution conditions, i.e., CH_3_CN/H_2_O containing K_2_CO_3_, was good, as it provided 57% RCY of **[^18^F]2a** compared to [^18^F]fluoride eluted with MeOH or MeOH/H_2_O containing K_2_CO_3_, which produced an RCY of 40% and 3%, respectively. This comparative study more clearly shows the differences in RCY between the reaction starting from [^18^F]fluoride after elution with CH_3_CN/H_2_O and subsequent azeotropic drying and the same reaction starting from [^18^F]fluoride after elution with MeOH without azeotropic drying. As mentioned above, it is tempting to speculate that the decrease in RCY from 57% to 40% could be due to the remaining water content in the reaction mixture. Considering the important advantage of TBMA-I that no azeotropic drying is needed to obtain an adequate RCY, we turned our attention to study aromatic radiofluorinations using TBMA-I as the PTC.

**Figure 2:**
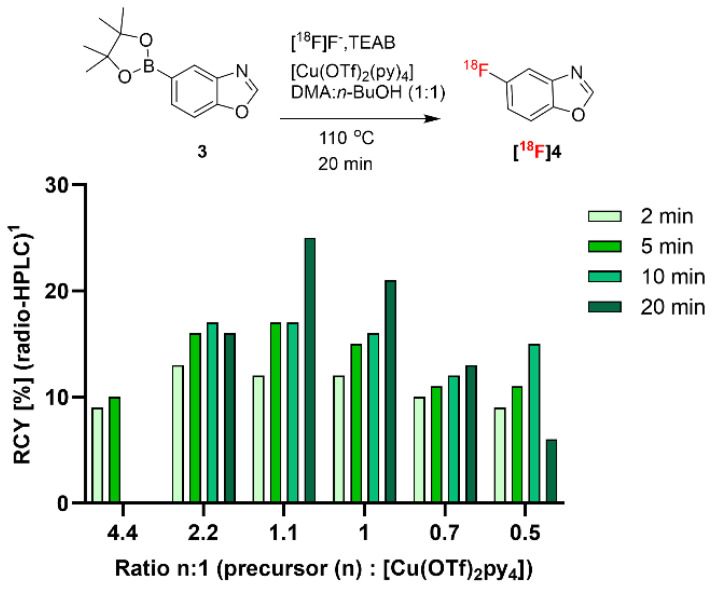
Optimization of radiofluorination under variation of the ratio of precursor **3** to Cu(OTf)_2_py_4_. ^1^ RCYs were determined by radio-high-performance liquid chromatography (radio-HPLC).

In a first step, starting from the reported reaction conditions of the copper-mediated aromatic ^18^F substitution of aryl boronic esters utilizing protic solvents in combination with polar aprotic solvents for optimal RCY [15], we adopted and optimized the precursor-to-[Cu(OTf)_2_py_4_] ratio for the ^18^F-labeling of 5-benzoxazole boronic acid pinacol ester (**3**), which served as precursor for the radiosynthesis of 5-[^18^F]fluorobenzoxazole (**[^18^F]4**; Figure 2). Figure 2 shows the optimization approach on the **3**-to-[Cu(OTf)_2_py_4_] ratio, revealing that the reported ratio of 2.2 equivalents provided 16% RCY of **[^18^F]4** after 20 min. Interestingly, in the case of precursor **3**, the ratio of 1.1:1 provided a maximum RCY of 25%. When increasing the ratio to 4.4, the product could not be detected after 10 min and a further decrease in the precursor amount compared to [Cu(OTf)_2_py_4_] did not improve the RCY. The optimal reaction condition employed a total reaction volume of 1.2 mL (DMA/*n*-BuOH, 1:1), and precursor **3** and [Cu(OTf)_2_py_4_] were added in concentrations of 25 mM and 22.5 mM, respectively.

**Figure 3:**
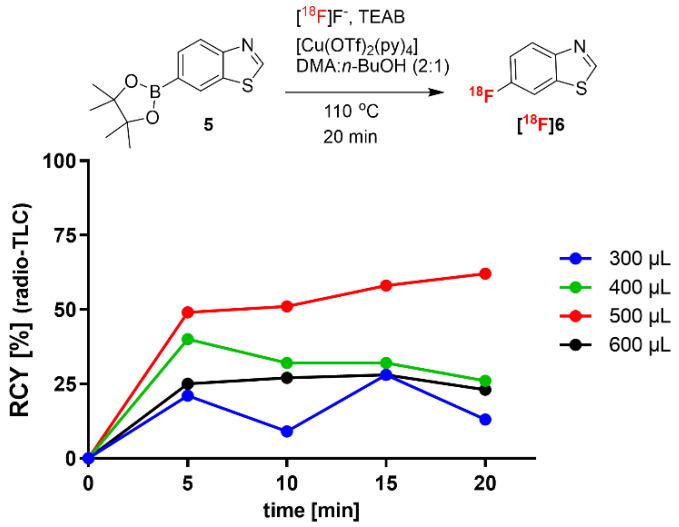
Optimization of radiofluorination on precursor **5** under reduction of the reaction volume.

In a second step, we aimed to minimize the reaction volume, as a reduced total amount of boronic acid ester precursor could simplify the purification of ^18^F-labeled compounds. In Figure 3, the effect of a reduced reaction volume on the ^18^F-fluorination of 6-benzothiazole boronic acid pinacol ester (**5**) is shown. Elution of [^18^F]fluoride with TEAB in MeOH allowed a reduction in the reaction volume to 300–600 µL, while the concentration of precursor **5** and [Cu(OTf)_2_py_4_] was kept constant (32.2 mM and 29.2 mM, respectively). The highest RCY achieved was 62% for 6-[^18^F]fluorobenzothiazole (**[^18^F]6**) after 20 min in a reaction volume of 500 µL, whereas further decrease or increase led to reduced RCYs. The found optimal conditions of the precursor-to-Cu-catalyst ratio (1.1:1), i.e., 16.1 µmol precursor and 14.6 µmol of [Cu(OTf)_2_py_4_], and a reaction volume of 500 µL, were then applied in the following experiments using TBMA-I as PTC.

Table 4 summarizes the direct comparison of TEAB and TBMA-I as the PTC in nucleophilic S_N_Ar, applying ^18^F-fluorination on 5-indoleboronic acid pinacol ester (**7**) as a model compound. We evaluated the reactivity of **7** using a total volume of 500 µL of DMA/*n*-BuOH (2:1) in the presence of TBMA as PTC, producing the desired ^18^F-fluorinated indole **[^18^F]8** with a 55–60% RCY. To compare our results, we performed the same reaction using TEAB as the PTC, which resulted in 10% more RCY of **[^18^F]8** than using TBMA-I as the PTC.

**Figure 4:**
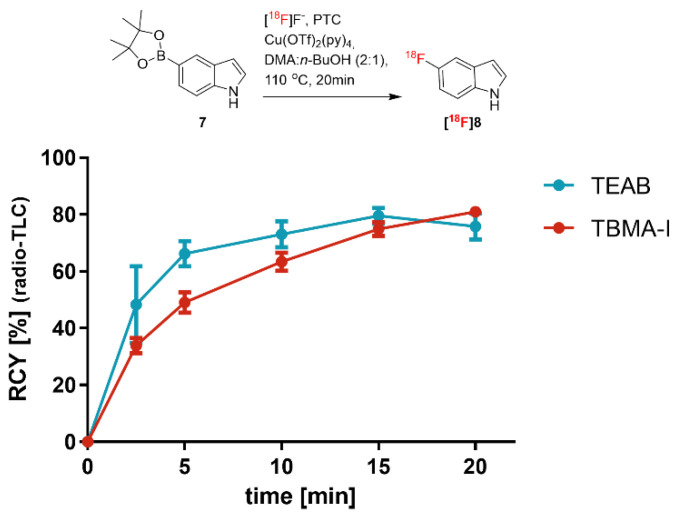
Time dependency of the radiosynthesis of 5-[^18^F]fluoro-1H-indole (**[^18^F]8**) using two different PTCs: TEAB versus TBMA-I. RCYs were determined by radio-TLC (*n* = 3).

Figure 4 depicts the time dependency of the RCY of **[^18^F]8** in the presence of either TEAB or TBMA-I. The aromatic ^18^F substitution using TBMA was initially (up to the reaction time of 2.5 min) about four times slower compared to the reaction using TEAB. The time course of the reaction clearly indicates that the TBMA-^18^F intermediate is more stable than the TEAB analog and less reactive, but reaches a comparable RCY of about 80% at 20 min. We suggest that hydrogen bonding between [^18^F]fluoride and *tert*-OH of TBMA tightly coordinates the [^18^F]fluoride. This could be the reason for a relatively slow S_N_Ar reaction due to the reduced availability of free fluoride anions in the reaction mixture.

**Figure 5:**
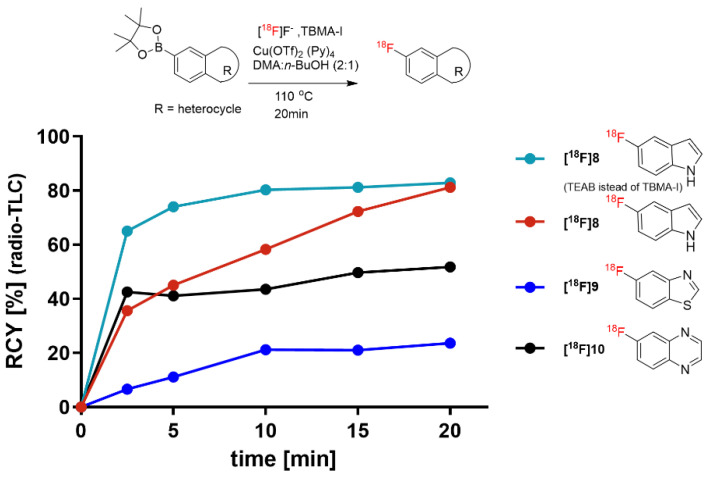
Radiosyntheses of various ^18^F-labeled aryl heterocycles from aryl boronic acid pinacol esters (reaction conditions: 16.1 µmol precursor, 14.6 µmol [Cu(OTf)_2_py_4_] in a reaction volume of 500 µL).

The applicability of TBMA-I as the PTC was further studied with various aryl pinacol boronic esters. Figure 5 shows the reactivity of TBMA-^18^F with commercially available boronic acid pinacol ester derivatives of 5-indole, 6-quinoxaline, and 5-benzothiazole. The found optimal reaction conditions were applied, affording the highest RCY of 80% for the indole **[^18^F]8**. The reactivity of TBMA-^18^F with 6-quinoxaline boronic acid pinacol ester and 5-benzothiazole boronic acid pinacol ester was lower, providing 57% and 29% RCY, respectively, of the corresponding ^18^F-fluorinated products after 20 min.

## 3. Materials and Methods

### 3.1. General

Radio-HPLC was performed on an Agilent 1100 system (Agilent Technologies, Böblingen, Germany) with a quaternary pump and variable wavelength detector and radio-HPLC detector HERM LB 500 (Berthold Technologies, Bad Wildbad, Germany) on a Chromolith RP-18e column (RP, 100 × 4.6 mm, 5 µm particle size, flow rate: 4 mL/min) using a linear gradient from 10–100% acetonitrile (0.1% TFA) in water (0.1% TFA) over 5 min. All ^18^F-labeled compounds were identified via the retention time (t_R_) of their non-radioactive reference compounds.

The reference compound **2a** was purchased from Santa Cruz Biotechnology (Dallas, TX, USA); **1**, **2b**, 6-fluorobenzothiazole (**6**), and 5-indoleboronic acid pinacol ester (**7**) were purchased from Sigma-Aldrich (Taufkirchen, Germany). Compound 5-benzoxazole boronic acid pinacol ester (**3**) was obtained from ABCR (Karlsruhe, Germany) and Fluorochem Ltd. (Hadfield, UK). The boronic acid pinacol ester derivatives of 6-benzothiazole (**5**), 6-quinoxaline, and 5-benzothiazole and the fluoro-substituted reference compounds 5-fluoro-1,3-benzoxazole (**4**), 5-fluorobenzothiazole (**9**) and 6-fluoroquinoxaline (**10**) were purchased from ABCR (Karlsruhe, Germany). Compound 5-fluoroindole (**8**) was purchased from Thermo Fisher (Alfa Aesar; Kandel, Germany).

No-carrier-added [^18^F]fluoride was produced through the ^18^O(p,n)^18^F reaction on a PETtrace 800 cyclotron (General Electric, Uppsala, Sweden) using H_2_[^18^O]O as the target at the Universitätsklinikum Würzburg (Klinik und Poliklinik für Nuklearmedizin, Experimentelle Nuklearmedizin, Radiopharmazie/PET-Zentrum, Samuel Samnick) and provided for this project.

### 3.2. Tri-Tert-Butanol Methylamine Iodide Salt (TBMA-I)

Tri-*tert*-butanolamine [(tri-*tert*-BuOH)A] (3.3 mmol) and methyliodide (1.0 mmol) were added to a sealed tube vial containing a stirring bar. The reaction mixture was stirred at room temperature for 12 h, then heated at 70 °C for 3 days. The removal of volatile compounds at high vacuum produced the desired product TBMA-I as a colorless liquid, ^1^H-NMR (400 MHz, CD_3_CN) δ 1.30 (s, 18H), 3.26, (s, 6H), 3.42 (s, OH), 3.51 (s, 3H), 4.00 (br, OH); ^13^C NMR (100 MHz, CD_3_CN) δ 31.12, 55.74, 69.67, 74.38.

### 3.3. Aliphatic ^18^F-Fluorination: Radiosynthesis of **[^18^F]2a**

[^18^F]fluoride was eluted from a Sep-Pak^®^ Light (46 mg) Accell^TM^ Plus QMA carbonate cartridge with as solution of Kryptofix^®^2.2.2 or TBMA-I, K_2_CO_3_ (1 M, 15 µL) in H_2_O (165 µL) and CH_3_CN (800 µL). The solvent was evaporated using a stream of argon at 85 °C and was co-evaporated to dryness with anhydrous CH_3_CN (3 × 500 µL). The precursor **1** (5.7 mg, 15.0 µmol) in anhydrous CH_3_CN (500 µL) was added and the reaction mixture was stirred at 85 °C for 20 min. Samples of reaction mixture were collected in 5 min intervals and the RCY was analyzed by radio-TLC (R_f_ = 0.9) and radio-HPLC (**[^18^F]2a**, t_R_ = 2.97 min).

### 3.4. Aromatic ^18^F-Fluorination: Radiosynthesis of **[^18^F]4**

The elution of [^18^F]fluoride was modified from the reported procedure of Zischler et al. [15]: After loading aqueous [^18^F]fluoride onto Sep-Pak^®^ Light (46 mg) Accell^TM^ Plus QMA carbonate cartridge from the male side, acetone (dry, 2.0 mL) was passed through the cartridge from the same side. Subsequently, air (10 mL) was applied from the male side and [^18^F]fluoride could be eluted directly into the reaction vial, using a solution of TEAB in *n*-BuOH (35.5 mM, 600 µL). A solution of 5-benzoxazole boronic acid pinacol ester (**3**, 7.4 mg, 30.0 µmol) and [Cu(OTf)_2_(py)_4_] (17.5 mg, 27.0 µmol) in DMA (600 µL) was pre-placed and brought to reaction with [^18^F]fluoride at 110 °C under air and stirring for 20 min. The reaction was monitored over 20 min by radio-HPLC (**[^18^F]4**, t_R_ = 1.77 min).

### 3.5. Aromatic ^18^F-Fluorination: Radiosynthesis of **[^18^F]6**

[^18^F]fluoride was eluted according to the reported procedure of Zlatopolskiy et al. [22]: [^18^F]fluoride was eluted directly into the reaction vial, using a methanolic solution of TEAB (20.9 mM, 500 µL) and MeOH was removed at 85 °C under a stream of helium. 6-Benzothiazole boronic acid pinacol ester (**5**, 4.2 mg, 16.1 µmol) and [Cu(OTf)_2_(py)_4_] (9.9 mg, 14.6 µmol) in solution with DMA:*n*-BuOH (2:1, 500 µL) was added and the reaction was stirred at 110 °C for 20 min under air. The reaction was monitored over 20 min by radio-HPLC (**[^18^F]6**, t_R_ = 2.27 min).

### 3.6. Aromatic ^18^F-Fluorination: Radiosynthesis of **[^18^F]8**

[^18^F]fluoride was eluted from a QMA cartridge with a solution of TBMA-I (3.9 mg, 10.4 µmol) in MeOH. Subsequently, the solution of the boronic ester precursor **7** (3.9 mg, 16.1 µmol) and [Cu(OTf)_2_(py)_4_] (9.8 mg, 14.6 µmol) in DMA/n-BuOH (2:1, 500 µL) was added. The reaction mixture was heated at 110 °C for 20 min under air. The reaction progress was monitored by sampling at intervals of 5 min by radio-TLC (R_f_ = 0.95) and radio-HPLC (**[^18^F]8**, t_R_ = 2.52 min).

Compounds **[^18^F]9** and **[^18^F]10** were synthesized according to **[^18^F]8** from the corresponding pinacol boronic ester precursors; the afforded products **[^18^F]9** and **[^18^F]10** were analyzed by radio-TLC (R_f_ = 0.84 and 0.76, respectively) and radio-HPLC (t_R_ = 2.21 min and 1.84 min, respectively).

## 4. Conclusions

In conclusion, we introduced the new *tert*-OH-functionalized quaternary ammonium salt tri-(*tert*-butanol)-methylammonium iodide (TBMA-I) for efficient [^18^F]fluoride elution and as the PTC in nucleophilic ^18^F-fluorination on aliphatic tosylates and aryl pinacol boronic ester precursors. TBMA-I showed promising PTC properties for its use in ^18^F chemistry, which includes efficient solubility in CH_3_CN, MeOH, and H_2_O; high recovery of [^18^F]fluoride from QMA carbonate cartridges applying MeOH; as well as decreased basicity compared to the Kryptofix^®^2.2.2-carbonate system. The coordination of [^18^F]fluoride with TBMA prevented the occurrence of hydrolytic byproducts during the aliphatic radiofluorination of bistosylates used as precursors in the ^18^F-synthesis of prosthetic groups. Moreover, TBMA-I also demonstrated its potential for use as a PTC in aromatic radiofluorination of aryl boronic acid pinacol esters.

## Figures and Tables

**Table 1:** Efficiency of ^18^F-elution from QMA cartridges using *tert*-alcohol functionalized amines as PTCs.

Entry	PTC (39.0 µmol)	Elution Mixture/Solvent (1 mL) ^1^	Recovery of [^18^F]fluoride (%)
1	-	CH_3_CN	3.3
2	Tri-(*t*-BuOH)A	CH_3_CN	11.1
3	TBMA-I	CH_3_CN	98.8
4	TBMA-I (19.5)	CH_3_CN	94.9
5	TBMA-I (78)	CH_3_CN	96.0
6	TBMA-I	MeOH ^2^ (0.5)	97.2
7	TBMA-I (10.4)	MeOH ^2^ (0.5)	98.2
8	TBMA-I (10.4)	*tert*-BuOH ^2^	70.1

^1^ Elution mixture: K_2_CO_3_ (1 M, 15 µL) and H_2_O (185 µL) in CH_3_CN (800 µL). ^2^ Without elution mixture.

**Table 2:** Reactivity of *tert*-alcohol-functionalized amines as PTCs for the ^18^F-substitution of 1,3-ditosylpropane (**1**) using various reaction solvents ^1^.

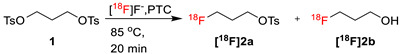					
Entry	PTC	Recovery of [^18^F]fluoride (%)	Solvent (0.5 mL)	RCY (%) (Radio-TLC) ^2^	
				[^18^F]2a	[^18^F]2b
1	Tri-(t-BuOH)A	11.1	CH_3_CN	-	-
2	Kryptofix^®^2.2.2	99.6	CH_3_CN	65	29
3	TEAB	98.3	CH_3_CN	60	7
4	TBMA-I	98.8	CH_3_CN	21	-
5	TBMA-I	99.6	tert-BuOH (1)/CH_3_CN (4)	50	
6	TBMA-I	92.3	*tert*-BuOH (0.5)/CH_3_CN (4.5)	57	-
7	TBMA-I	99.4	DMSO	7	-
8	TBMA-I	96.9	DMF	2	-

^1^ Unless noted otherwise, all reactions were carried out using 15.0 µmol of K_2_CO_3_ (1 M, 15 µL) and 39.0 µmol of PTC at 85 °C for 20 min. ^2^ Radio-thin layered silica gel chromatography (radio-TLC) plates were run with EtOAc mobile phase; the shown values are the mean of three determinations.

**Table 3:** Radiofluorination of **1** with TBMA-[^18^F]fluoride eluted with MeOH ^1^.

Entry	PTC (10.4 µmol)	Recovery of [^18^F]fluoride (%)	Solvent (0.5 mL)	RCY (%) of [^18^F]2a (Radio-TLC)
1	TBMA-I	98.3	*tert*-BuOH (0.5)/CH_3_CN (4.5)	40
2	TBMA-I	98.3	*tert*-BuOH (1)/CH_3_CN (4)	25
3	TBMA-I	97.1	*tert*-BuOH (4)/CH_3_CN (1)	8
4 ^2^	TBMA-I	98.6	*tert*-BuOH (0.5)/CH_3_CN (4.5)	3
5	TBMA-I	93.7	CH_3_CN	14

^1^ Elution with MeOH, no azeotropic drying step. ^2^ Addition of 15 µL aq K_2_CO_3_ (1 M) in reaction.

**Table 4:** Radiosynthesis of 5-[^18^F]fluoro-1H-indole (**[^18^F]8**) from 5-indoleboronic acid pinacol ester (**7**) in the presence of TEAB or TBMA-I ^1^.

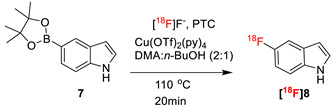					
Entry	Elution Solvent (0.5 mL)	PTC (µmol)	Recovery of [^18^F]fluoride (%)	RCY (%) of [^18^F]8	
				Radio-TLC	Radio-HPLC
1	MeOH	TBMA-I (10.4)	96.4	55	60
2	MeOH	TEAB (10.4)	95.8	65	78

^1^ All reactions were carried out with 16.1 µmol of **7**, 14.6 µmol of Cu(OTf)_2_(py)_4_ in a reaction volume of 0.5 mL (DMA/n-BuOH, 2:1 *v*/*v*).

## Data Availability

All data are contained within the article.
